# Comparison of the effects of platelet-rich or growth factor-rich plasma on intestinal anastomosis healing in pigs

**DOI:** 10.1186/s12917-017-1102-8

**Published:** 2017-06-19

**Authors:** Gessica Giusto, Cristina Vercelli, Selina Iussich, Massimiliano Tursi, Giovanni Perona, Marco Gandini

**Affiliations:** Department of Veterinary Sciences, University of Turin, Largo Paolo Braccini, n. 2, Grugliasco, 10095 Turin, Italy

**Keywords:** Platelet rich plasma, Anastomosis, Leakage, Pig

## Abstract

**Background:**

The use of autologous platelet-rich plasma (PRP) and plasma rich in growth factors (PRGF) has been proposed for the treatment of several acute and chronic syndromes, such as corneal epithelial defects and dry eye syndrome, gum bleeding during oral surgery, and in orthopaedic surgery. We hypothesized that PRGF, rather than PRP, could be more effective because of its intrinsic characteristics in promoting the healing of intestinal anastomosis. The purpose of the present study was to evaluate and compare the effects of PRP and PRGF on various parameters of anastomotic healing in a swine model.

**Methods:**

Eight female pigs were randomly assigned to two groups and subjected to hand sewn jeujuno-jejunal appositional extramucosal anastomoses. For each animal, a total of six anastomoses were performed: two were considered controls and received no treatment, while the remaining four anastomoses were treated with PRP or PRGF of which both were prepared at a platelet concentration that was respectively 3.4-fold and 2.81-fold higher than the original platelet count. In each animal, either PRP or PRGF was used as a treatment, to avoid interference among products. Animals were euthanized after 8 days and the anastomoses were evaluated and compared for the presence of adhesions, anastomotic leakage, bursting pressure, and histological appearance.

**Results:**

The concentration of platelets in PRP was 3.41-fold higher (range, 3.20–4.24) that the concentration in whole blood, while the concentration in PRGF was 2.81-fold higher (range, 2.89–4.88).

The results obtained from the present study highlighted that there are no differences between anastomotic samples treated with either PRP or PRGF preparations, except for a significant increase in epithelization of the intestinal mucosa at the anastomotic site in the PRGF group.

**Conclusions:**

Both PRP and PRGF suspensions should be considered a safe strategy and represent a relatively low-cost technology that is flexible enough to be applied in several therapeutic fields. No true benefit could be proven in our study compared to the no treatment following anastomoses formation, with the exception of enhanced epithelization of the mucosa in the PRGF group.

**Electronic supplementary material:**

The online version of this article (doi:10.1186/s12917-017-1102-8) contains supplementary material, which is available to authorized users.

## Background

Surgical techniques are continuously evolving to be more efficient for patients and to reduce the duration of post-operative recovery. In gastrointestinal surgery, a major challenge is represented by anastomosis dehiscence [1]. Recently, several studies have focused on identifying a strategy to reduce this complication, which represents a failure in the surgical procedure that can be potentially fatal [2]. Some of these investigations have suggested using substances that are able to accelerate the wound healing process. Regenerative medicine may offer some important guidance in this area [3]. The use of platelet-rich plasma (PRP) alone or in combination with growth factors (preparations rich in growth factors, PRGF) [4] naturally secreted by platelets, such as platelet-derived growth factor (PDGF), transforming growth factor-β (TGF-β), and vascular endothelial growth factor (VEGF), may play a role in improving anastomotic healing [3]. PRP and PRGF differ in composition and methods of preparation. Several methods to produce a biologically active product have been developed and they differ in the concentration of growth factors, white blood cells, and characteristics of the fibrin scaffold [5, 6]. PRP was defined by Ehrenfest et al. [7] as a preparation with leucocytes and with a low-density fibrin network after activation. PRGF contains moderate platelet concentration, no leukocytes, nor a three dimensional fibrin scaffold [5, 6]. From a therapeutic perspective, there are two main advantages in using PRGF. First is the release of proteins and growth factors from platelets that stimulate regeneration. Second is the creation of a three-dimensional fibrin matrix that retains and releases growth factors and acts as a temporal scaffold for the cells [8]. Furthermore, the absence of leukocytes is an important characteristic of PRGF. Leukocytes produce metalloproteinase, free radicals, reactive oxygen species, and nitrogen, which can cause damge to healing tissues [8]. The rationale for PRP therapy lies in reversing the blood ratio by reducing the amount of red blood cells, which are less useful in the healing process, to approximately 5% and to increase the amount of platelets to 94% to stimulate recovery [5]. The use of autologous PRP and PRGF has been proposed for the treatment of several acute and chronic syndromes, such as for corneal epithelial defects and dry eye syndrome, avoiding gum bleeding during oral surgery, and in orthopaedic surgery [4, 9–12]. The most important feature of PRP is that autologous products circumvent immunogenic reactions and disease transmission. According to Dr. Anitua, who first proposed the technique, PRGF has some peculiar characteristics that may favour its use over that of PRP. In particular, the lower number of leucocytes in the PRGF may improve healing because of the absence of inflammatory cells [13].

With each passing year, the number of relevant articles published in PubMed has increased (accessed October 13, 2015); however it is difficult to compare all published results, given the lack of uniformity in processing platelets and the different techniques used. Papers dealing with the possible use of PRP have not been in complete agreement. The majority suggest that these substances have a positive effect on wound healing of colonic anastomosis, while one paper [3] indicated that application of PRP could only increase fibrosis and granulation tissue, without improving the breaking strength of anastomotic sites. To date, all studies have dealt with PRP, while none have tested the use of PRGF on anastomosis healing.

Our hypothesis was that PRGF, rather than PRP, could be more effective in promoting the healing of intestinal anastomosis because of its intrinsic characteristics (i.e., low number of leukocytes). The purpose of the present study was to evaluate and compare the effects of PRP and PRGF on various parameters of anastomotic healing in a swine model.

## Results

### Assessment of the characteristics of autologous preparations

Results from assessing the characteristics of the autologous preparations are reported in Table 1. The concentration of platelets and leukocytes of freshly collected whole blood for both groups were within the normal range of values for Landrace pigs (platelets 217–770 × 10^3^/mL, leukocytes 7–20 × 10^3^/mL) (http://www.ahc.umn.edu/rar/refvalues.html, accessed 15-06-2017). The concentration of platelets in PRP was 3.41-fold higher (range, 3.20–4.24) than the concentration of whole blood, while in PRGF, it was 2.81-fold higher (range, 2.89–4.88).

**Table 1:** Comparison of the characteristics of the preparations (median-range)

	Group PRP			Group PRGF		
	Whole blood (cells ×10^3^/ μL)	PRP (cells ×10^3^/ μL)	Prep/WB ratio	Whole blood (cells ×10^3^/ μL)	PRGF (cells ×10^3^/ μL)	Prep/WB ratio
Platelet count	387 (295–622)	1321 (945–2641)	3.4 (2.5–4.9)	376 (285–495)	1565 (825–2420)	3.4 (1.5–5.3)
Leukocyte Count	13.76 (11.5–17)	1.72^a^ (0.8-3.5)	0.132^b^ (0.05-0.20)	13.85 (10.2–16)	0.17^a^ (0.01-1.020)	0.02^b^ (0.001-0.67)

^a^ *P* = 0.0007

^b^ *P* = 0.0002

### Surgical procedures

All animals recovered uneventfully from anaesthesia and there were no surgery-related complications in the postoperative period

### Macroscopic examination

**Fig. 1:**
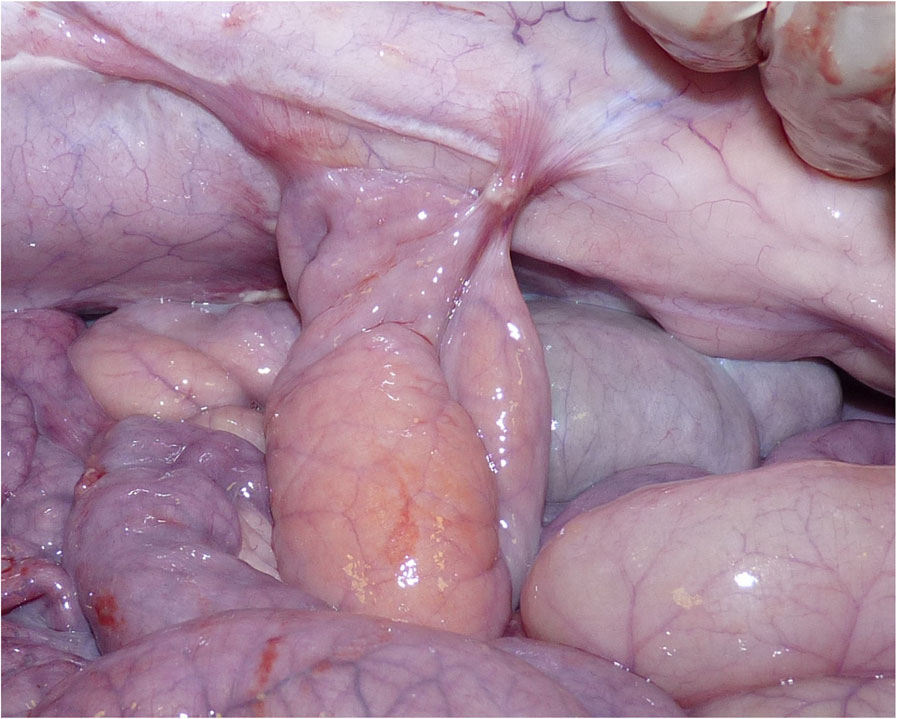
A grade 3 adhesion in the PRP group

Macroscopic evaluation showed no evidence of leaking in any anastomoses in either the control or treated animals. Anastomoses treated with PRP showed more adhesion formation (10/16) compared to those treated with PRGF (5/16) or controls (6/16); nevertheless, this increase in adhesion formation was not significantly different (*P* = 0.169). When adhesion formation was noted, it was scored 1 or 2, with only one case in the PRP group that was scored 3 (Fig. 1). The median adhesion score for group PRP was 1 (0–3) and 0 for group PRGF (0–2).

### Bursting pressure

Bowel sections treated with PRGF demonstrated more resistance at the bursting pressure test (Table 2), although not significantly different from controls and PRP-treated anastomoses. PRP-treated anastomoses were significantly less resistant than intact intestinal tissue.

**Table 2:** Bursting pressure measurements (median-range)

	PRP-treated anastomoses	PRGF-treated anastomoses	Control anastomoses	Intact bowel
Bursting pressure	117.5 (80–190)^a^	165 (100–190)	154.0 (50–180)	175 (160–190)^a^

^a^ *P* = 0.0007

### Histology

**Fig. 2:**
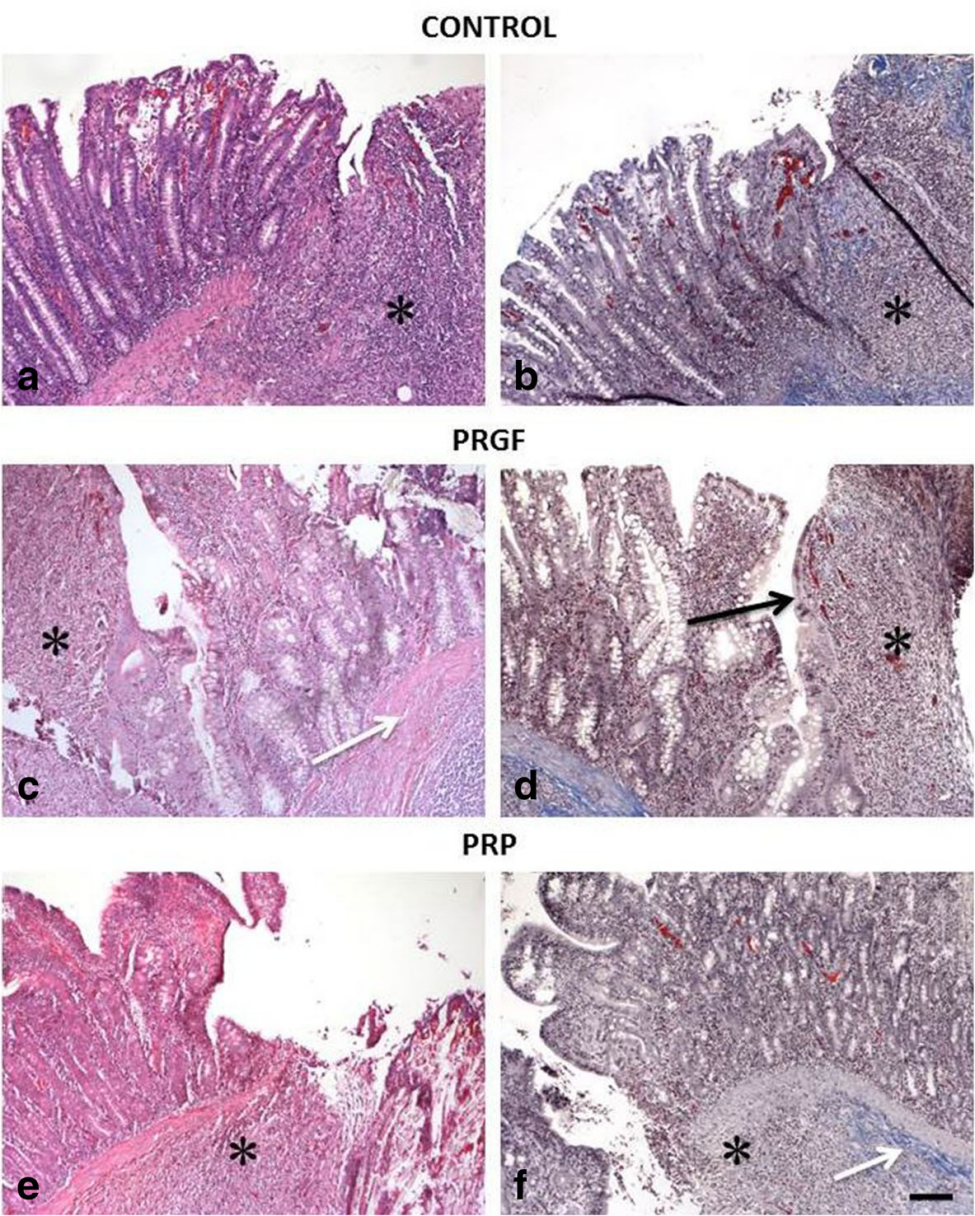
Histological appearance of the anastomotic site in each group: **a**,**b** CONTROL. **c**,**d** PRGF. **e**,**f** PRP, Bar: 100 μ, **a**, **c**, **e** Hematoxylin and eosin 10×. **b**, **d**, **f** trichrome Masson staining 10×, *Inflammation, *Black Arrow*: epithelization, *White arrow*: fibrosis

Results of the histological evaluation are reported in Table 3. There was no significant difference among the samples treated with PRP, PRGF, and controls relative to inflammatory infiltrate, proliferation of fibroblasts, neovascularisation, or deposition of collagen (Fig. 2).

**Table 3:** Results of the histological evaluation (median-range)

	PRGF	PRP	Control	*p* value
Epithelization	2.5 (0–3)^a,b^	1 (0–2)^a^	0.5 (0–2)^b^	a = 0.0012 b = 0.0005
Inflammation	3 (1–3)	2.5 (2–3)	3 (1–3)	0.668
Fibrosis	2 (1–3)	3 (1–3)	3 (1–3)	0.135
Neovascularization	1 (0–2)	2 (1–3)	2 (1–3)	0.079
Collagen	2.5 (1–3)	2 (1–3)	2 (1–3)	0.971

Microscopic evaluation highlighted a significant increase in epithelization of the mucosa in the PRGF-treated group (Table 3)

## Discussion

During the early stages of intestinal wound healing, platelets play an important role during the initial 72 h after injury [14]. The release of growth factors from platelets mediates the healing process [15]. The use of autologous substances, such as platelet rich products, has been considered a promising advance for new surgical and clinical approaches. Furthermore, recent advances in their use should eliminate the risk of immunological reactions. Moreover, it is assumed that they increase the local growth factor concentrations at the site of healing, thereby accelerating the wound healing process [15]. This type of biological treatment mimics natural tissue healing, while optimising and reducing the time required [5]. Thus, all proteins necessary for tissue repair are released locally.

To the best of our knowledge, this is the first study comparing PRP and PRGF healing effects on intestinal anastomosis.

Bursting pressure is considered to be a more accurate measure as it reflects the physiologic strain in intestinal tissue rather than than the breaking strength [15]. Intestinal healing is characterised by three phases of healing: inflammatory, proliferative, and the maturation phases. These steps occur between post-operative days 0 and 4, from days 3 to 14, and from days 10 to 180, respectively [15]. Usually, during the first phase, fibrin contributes to wound healing and strength, but the major strain is allocated to the sutures. In a normal setting, between days 3 and 4, the anastomotic strength is lower due to fibrinolysis and collagen deposition. Under such conditions, dehiscence of the suture line can easily occur. This study considered only the proliferative phase because all pigs were euthanized at day 8 after surgery. We specifically took into consideration this period because, in this phase, macrophages are involved in fibrin debridement (occurring in the inflammatory phase) and natural growth factor production is at its maximum peak and might modulate fibrosis and angiogenesis [16]. The application of a PRP or a PRGF treatment should promote intestinal healing and lower the risk of dehiscence.

The results obtained from the present study highlighted that there are no significant differences between anastomotic samples treated with either PRP or PRGF preparations. Bursting pressure showed a high resistance of PRGF-treated anastomoses, in comparison with PRP treated ones. A statistical significance was found between the PRP-treated anastomoses and the healthy intestine. This finding could be due to the presence of leukocytes in the PRP, which release substances (such as metalloproteinases) that may damage healing tissues. Leukocytes, by participating in an inflammatory cascade, could decrease anastomotic bursting pressure more so than in the control anastomoses, where leukocytes concentration is lower. During the proliferative phase, an increase in neo-vascularisation, fibroblast proliferation, and collagen deposition should be noted along with a decrease in inflammation. However, in our study, no difference was found between groups regarding these parameters. Our results showed only a better epithelization of the jejunal mucosa in the PRGF-treated group. The data from this study are partially in accordance with the results of Fresno et al. [3]. The authors employed a similar experimental design, but, following treatment of the anastomosis with the PRP suspension, the anastomotic sites were covered with omentum. This could cause an increase in fibrosis at the anastomotic sites that could be considered a bias. In the present study, in order to avoid this type of interference, the omentum was not placed on the anastomosis site.

In our study anastomoses treated with PRP showed more adhesion formation compared to samples treated with PRGF or controls. These results could be explained by the composition of the PRP, which contains leukocytes and platelets. Adhesion formation is triggered by inflammatory mediators and by dispersion of fibrin onto the affected surface [17]. Converseley, as reported by Anitua et al. [6], who first proposed the technique, PRGF was found to not contain any leukocytes. Instead, PRGF was found to contain a moderate platelet concentration, which would circumvent all potential pro-inflammatory effects that could explain the higher number of adhesions in the PRP group.

As already argued by Fresno et al. [3], the immersion of the intestinal edges in the treatment bath may present some issues. Intestinal edges after a resection usually bleed, and may be contaminated with intestinal contents, thus potentially influencing PRP or PRGF efficiency and concentration.

Some authors [18, 19] have investigated platelet rich plasma and considered eliminating leukocytes from such preparation so as to avoid higher concentrations of pro-inflammatory cytokines that might reduce tissue healing. The study by Anitua et al. [12] used PRP that was either rich or poor in leucocytes in order to evaluate whether this could influence tissue healing; however, it appeared that leucocyte concentrations did not interfere with wound healing after oral surgery. Some issues were raised by Del Buono and colleagues [20] against the indiscriminate use of PRP or PRGF. They explained that these substances could successfully improve wound healing in orthopaedic and oral surgery, but should be carefully handled for other applications, because concentrations of PRP or PRGF, doses, timing and length of applications have not been defined yet. Yamaguchi et al. [15] investigated different concentrations of PRP to evaluate dose-specific effects on intestinal anastomosis in rats and established that the optimal concentration of platelets should be around 2.5-fold higher than that found in the initial blood collection. As also reported by Yamaguchi et al. [15], clinicians should be cautious in thinking “if some is good, more is better” as it does not apply to PRP applications. It has been shown that platelet concentrations of 4.5–5.5-fold higher than that found in whole blood may interfere with the normal wound healing process [14, 21]. In our study, platelet concentrations achieved in both groups (PRGF and PRP) were 3.4- and 3.5-fold higher, respectively, than the initial ratio. This is higher than what is considered the optimal ratio to achieve a maximum effect from plasma-rich preparations on intestinal anastomosis [15] and perhaps this could be the cause of the poor effect demonstrated in our study. Reducing the platelet concentration to around 2.5-times the plasma concentration or changing the method of its application at the anastomotic site may yeld different results.

Fresno and colleagues [3] did not find a significant difference in breaking strength when PRP was applied to anastomoses in pigs, but they found a higher degree of fibrosis and granulation tissue. While the finding on bursting pressure is in accordance with our study, the difference between their histological findings and ours may be due to a certain degree of subjectiveness in the evaluation. Yol and colleagues [22] instead found that PRP application increased the bursting strength of intestinal anastomoses in rats. In this case, the difference with our study may be due to differences in species (rat vs pig), intestinal segment (colon vs jejunum) and method of production of PRP.

Certainly, as in the study conducted by Fresno and colleagues, our study is limited from having tested the two treatments together with the control in the same animal. It is possible that this was a confounding factor and that, eventually, resulted in growth factors from one preparation effecting sites that were distant from the application site.

Further studies are needed to prove the effectiveness of platelet rich preparations in species other than rats and in different intestinal segments. Furhtermore, methods that could modulate the final concentration of platelets in the preparation would be beneficial.

## Conclusions

Both PRP and PRGF suspensions should be considered a safe strategy and represent a relatively low-cost technology that are flexible enough to be applied in several therapeutic fields. No true benefit could be proven in our study compared to the no treatment following anastomoses formation, with the exception of enhanced epithelization of the mucosa in the PRGF group. Nevertheless, further studies are required to clarify the molecular mechanisms underlying the biological effects on wound healing and to understand how to improve this promising tool.

## Methods

The study was approved by the Bioethical Committee of the University of Turin and by the Italian Ministry of Health.

### Animals

Eight female pigs (Landrace X Large White), weighing 40 to 48 kg, were kept in standard conditions and fed for 1 week before the experiment. Pigs were randomly assigned to two groups (PRP and PRGF) and subjected to hand sewn jejuuno-jejunal appositional extramucosal anastomoses [22]. For each animal, a total of six anastomoses were performed: two were considered controls and received no treatment, while the remaining four anastomoses were treated with PRP or PRGF, according to the assigned group. In each animal, either PRP or PRGF was used as a treatment, to avoid interference among products. This led to a total of 16 untreated anastomoses (control), 16 anastomoses treated with PRGF, and 16 treated with PRP.

### PRP preparation

For all pigs in the PRP group, blood samples were collected from the jugular vein before surgical procedures, just before induction of anaesthesia. For each pig, 5 mL blood samples were drawn into 12 different tubes containing buffered 3.8% sodium citrate. Blood samples were centrifuged at 786 g for 10 min. Next, supernatant and buffy coat were removed and the remaining sample was centrifugated at 526 g for 10 min. The resulting pellet consisted of platelets, which were harvested and re-suspended with 500 μL of supernatant. The suspension was then stored at 20 °C for a time ranging between 45 and 90 min before being applied to the anastomotic site. Immediately prior to the application at the anastomotic site, 1 mL of this suspension was transferred to a Petri dish and 50 μL of calcium chloride (25 mM) was added to activate the PRP [3].

### PRGF preparation

Similar to the pigs in the PRP group, blood was collected from all pigs in the PRGF group from the jugular vein just before induction of anaesthesia. For each pig, 5 mL blood samples were drawn into 12 different tubes containing buffered 3.8% sodium citrate. Samples were then centrifuged using a PRGF-endoret system^1^ at 580 g for 8 min. This procedure led to the separation of the different fractions: the first (Fraction 1: F1) containing a platelet count similar to that of peripheral blood, the second (F2) containing a higher quantity of platelets and growth factors, and the third (F3) containing the highest concentration of both platelets and growth factors compared to other fractions. F3 was the only fraction useful for the preparation of PRGF, while F1 and F2 were discarded [4]. Immediately prior to application at the anastomotic site, 1 mL of suspension, prepared as described above, was transferred to a Petri dish and 50 μL of 10% calcium chloride was added to activate the preparation [5].

For both PRP and PRGF suspensions, both platelet and white blood cell counts were obtained and compared.

### Group assignment

Animals and techniques were assigned randomly, using a random number generator (www.random.org).

### Assessment of the characteristics of the autologous preparation

The number of platelets and leukocytes in swine whole blood (before centrifugation) and in PRGF and PRP fractions was assessed using the Advia 120 Bayer Haematology Analyser^1.^ 2 Because each autologous preparation (PRP and PRGF) was obtained from animals of two groups, a ratio between the platelet (PLT) count in each preparation and the platelet count of the whole blood (WB) was calculated for each group for comparison purposes using the formula: PLT ratio = (PLT count in autologous preparation/PLT in whole blood). Similarly, the ratio between the number of leukocytes in the preparation and the number of leukocytes in the whole blood was obtained using the formula: Leukocyte ratio = Leucocyte count in autologous preparation/Leukocytes in whole blood.

In addition, the collection efficiency (%) was calculated as follows: (Preparation volume × preparation PLT count)/(Whole blood volume × Whole blood PLT count) × 100 [3] and was compared between groups.

### Surgical procedures

Twelve hours before surgery, animals were not allowed to consume food, but were allowed to consume water ad libitum. Pigs were pre-medicated with an intramuscular injection of xylazine^2^ 3 (2 mL/kg, intramuscularly [IM]) and anaesthesia was induced using an intramuscular injection of tiletamine and zolazepam^3^ 4 (4.4 mg/kg, intramuscularly [IM]). The trachea was intubated and anaesthesia was maintained with 2–2.5% isoflurane^4^ 5 in 100% oxygen and spontaneous ventilation, with a semi-closed circular anaesthetic system. Animals were placed in dorsal recumbency and the abdomen was shaved and aseptically prepared using 10% povidone iodine. After a midline laparotomy, the small intestine was exposed. At 30 cm distally from the Treitz’s ligament, six resections were performed on the jejunum, approximately 40 cm apart.

The methods by which resection and anastomosis formation were performed were standardized to limit the harvesting of tissue such that there would be no differences in vascularization. A wedge of tissue was obtained by cutting the intestine along two lines at an angle of 60° starting at the same mesenteric site. The anastomotic technique was the same for all anastomoses and was performed by a single surgeon (MG). Resection and anastomosis was performed extracorporeally and moist laparotomic gauzes were used to reduce surgical field contamination

Before anastomosing the two segments, the edges were dipped in the activated PRP or PRGF, according to the allocated group, and maintained for 5 min until the preparation formed a gelatinous clot [3]. The surgeon performing the anastomoses was blinded regarding the treatment. Being careful not to remove this clot, intestinal continuity was then restored with a jejuno-jejunal end-to-end anastomosis, using a single-layer continuous modified appositional extra-mucosal suture [22] with a Glycomer 631 USP 3–0^5.^ 6

As described above, two anastomoses in each subject were used as controls. The intestinal stumps were dipped in 0.9% sodium chloride solution for 5 min before application to the anastomosis sites.

No measures were undertaken to reduce the risk of adhesions, other than keeping the intestine moistened during the procedure and, in particular, the anastomotic sites were not covered with omentum. The mesentery was closed with a simple continuous suture with Glycomer 631 USP 3-0.^5, 6^

The abdomen was closed using a routine, mass simple continuous suture of the fascia with PDS USP 1^6^ 7 and with stapled closure of the skin using staples^6,7.^ 8 Animals were treated preoperatively with a single intramuscular administration of benzipenicillin-diistreptomicine^6,7,8^ 9 (20 mg/kg, intramuscularly [IM]), while post-operative analgesic therapy consisted of intramuscular buprenorphine^7,8,9^ 10 (0.01 mg/kg, intramuscularly [IM]) once and as needed based upon postoperative monitoring. During recovery, pigs were placed under an infrared heat lamp. After recovery, access to water and food was allowed after 6 and 18 h, respectively.

Postoperatively, pigs were monitored twice daily for fever and pain (which were determined by monitoring the animal’s appearance, food intake, activity, and response to wound palpation).

All animals were anaesthetised 8 days after surgery using an intramuscular injection of tiletamine and zolazepam^3,4^ (4.4 mg/kg, intramuscularly [IM]) and euthanized by intracardiac injection of embutramide, mebenzonium iodide, and tetracaine hydrochloride solution^8,9,10^ 11 ^.^ 12

### Postoperative period

All animals recovered uneventfully from anaesthesia. Only two pigs showed postoperative pain that was controlled by further doses of buprenorphine.

### Macroscopic examination

Necropsy was performed by an operator blinded to the techniques. During the necropsy, the following findings were recorded: a) adhesions; b) signs of peritonitis; c) anastomotic leakage; and d) presence of abscesses or granulomas at the anastomotic sites. The number of adhesions involving the anastomoses was determined and compared within groups.

The evaluation of adhesions in each anastomotic site was classified according to the scale proposed by Van der Ham et al. [23] and by Wang and colleagues [24] (Table 4).

**Table 4:** Grading of adhesions

Score	Description
0	No adhesion
1	Minimal adhesions, mainly between the anastomosis and omentum
2	Moderate adhesion, between omentum and anastomosis, or between the bowel and anastomosis
3	Severe and extended adhesions with possible formation of abscesses

### Bursting pressure

Directly after euthanasia and necropsy, tThe bursting pressure was measured using a modified inflation tank test [25, 26]. Intestine portions were closed with plastic tie-wraps that were placed 10 cm proximally and distally to the anastomotic site. At one end, a 20 G needle, attached to a column manometer, was tunnelled through the intestinal wall. Similarly, at the opposite end, another 20 G needle was inserted and attached to a syringe pump. The specimen was maintained in 0.9% warm saline as the syringe pump^11,12^ 13 inflated the tissue with air at a rate of 0.5 L/min. The procedure was digitally filmed. Anastomotic leakage and bursting were indicated by the presence of air bubbles and by a sudden pressure stop/drop, as measured by a manometer. The exact peak pressure was reported with the help of videography [25]. The bursting pressures of healthy intestinal samples distant from the anastomotic sites during necroscopy were also recorded as controls [27].

All samples were sectioned longitudinally on the antimesenteric margin and fixed in 10% buffered formalin.

### Histology

Bowel samples were fixed in 10% buffered formalin, embedded in paraffin, cut into 4-μm sections, stained with haematoxylin and eosin (H&E), and examined by two blinded expert pathologists to evaluate inflammation and neovascularisation. Sample slices were also stained with Masson’s trichrome to assess fibroblast proliferation. Each parameter (i.e., epithelialization/ulceration, inflammatory infiltrate, proliferation of fibroblasts, neovascularization, and deposition of collagen) was scored on a scale proposed by Erlich et al. and modified by Philips et al. [28, 29] (Table 5).

**Table 5:** Grading of histological parameters

Score	Description
0	No evidence
1	Occasional evidence
2	Light scattering
3	Abundant evidence

### Statistical analysis

Data are presented as median (range). Normality of data was determined by the Shapiro-Wilk test. To compare platelet and leukocyte counts, an unpaired Student’s *t*-test with Welch’s correction was used, while the Mann–Whitney test was employed to compare ratios. The Kruskal–Wallis test with Dunn’s multiple comparison tests was used to compare bursting pressures and histological parameters.

The number of adhesions per group was compared using the Chi square test, while the mean adhesion score for each group was compared using the Kruskal–Wallis test with Dunn’s post-hoc test. Data were analysed using GraphPad Prism 6.0 software^9^ software^12,13^ and a *P*-value <0.05 was considered significant.

## Additional file

Additional file 1: Cerficate of English Editing. (PDF 238 kb)

## Abbreviations

IM: Intramuscularly
PDGF: Platelet-derived growth factors
PLT: Platelet
PRGF: Preparations rich in growth factors
PRP: Platelet-rich plasma
TGF-β: Transforming growth factor-β
VEGF: Vascular endothelial growth factor
WB: Whole blood

## Footnotes

1 BTI Biotechnology Institut, Milano, Italy

2 Siemens AG, Wittelsbacherplatz, Munich, Germany

3 Bayer Animal Health, Monheim, Nordrhein-Westfalen, Germany

4 Virbac Italia, Milano, Italy

5 Esteve Italia spa, Milano, Italy

6 Covidien, Segrate Milano, Italy

7 Johnson and Johnson, Milano, Italy

8 Weck Visistat R35

9 Fatro, Ozzano dell’Emilia, Italy

10 Schering-Plough, Segrate Milano, Italy

11 Intervet Italia srl, Segrate Milano, Italy

12 Care Fusion, Firenze, Italy

13 GraphPad Software Inc., La Jolla, USA
