# Supplementary material for: Comparison of the effects of platelet-rich or growth factor-rich plasma on intestinal anastomosis healing in pigs
Source: BMC Vet Res. 2017 Jun 19;13:188. doi: 10.1186/s12917-017-1102-8 (PMC5477282; doi:10.1186/s12917-017-1102-8)
Supplement: Additional file 1: — Cerficate of English Editing. (PDF 238 kb) [file 12917_2017_1102_MOESM1_ESM.pdf]

CERTIFICATE OF ENGLISH EDITING

This document certifies that the paper listed below has been edited to ensure that the language is clear and free of errors. The edit was performed by professional editors at Editage, a division of Cactus Communications. The intent of the author's message was not altered in any way during the editing process. The quality of the edit has been guaranteed, with the assumption that our suggested changes have been accepted and have not been further altered without the knowledge of our editors.

Title Of The Paper

Comparison of the effects of platelet-rich or growth factor-rich plasma on intestinal anastomosis healing in pigs

Authors

Dr. Marco Gandini

Job Code

MANDI_11_2

Signature

Nikesh Gosalia,
Vice President, Author Services, Editage

Date of Issue

8th April 2017

Editage, a brand of Cactus Communications, offers professional English language editing and publication support services to authors engaged in over 500 areas of research. Through its community of experienced editors, which includes doctors, engineers, published scientists, and researchers with peer review experience, Editage has successfully helped authors get published in internationally reputed journals. Authors who work with Editage are guaranteed excellent language quality and timely delivery.
